# Burn Injury Reduces Neutrophil Directional Migration Speed in Microfluidic Devices

**DOI:** 10.1371/journal.pone.0011921

**Published:** 2010-07-30

**Authors:** Kathryn L. Butler, Vijayakrishnan Ambravaneswaran, Nitin Agrawal, Maryelizabeth Bilodeau, Mehmet Toner, Ronald G. Tompkins, Shawn Fagan, Daniel Irimia

**Affiliations:** 1 Surgery Department, Massachusetts General Hospital, Shriners Hospital for Children, and Harvard Medical School, Boston, Massachusetts, United States of America; 2 BioMEMS Resource Center, Center for Engineering in Medicine and Surgical Services, Massachusetts General Hospital, Shriners Hospital for Children, and Harvard Medical School, Boston, Massachusetts, United States of America; University of Milan-Bicocca, Italy

## Abstract

Thermal injury triggers a fulminant inflammatory cascade that heralds shock, end-organ failure, and ultimately sepsis and death. Emerging evidence points to a critical role for the innate immune system, and several studies had documented concurrent impairment in neutrophil chemotaxis with these post-burn inflammatory changes. While a few studies suggest that a link between neutrophil motility and patient mortality might exist, so far, cumbersome assays have prohibited exploration of the prognostic and diagnostic significance of chemotaxis after burn injury. To address this need, we developed a microfluidic device that is simple to operate and allows for precise and robust measurements of chemotaxis speed and persistence characteristics at single-cell resolution. Using this assay, we established a reference set of migration speed values for neutrophils from healthy subjects. Comparisons with samples from burn patients revealed impaired directional migration speed starting as early as 24 hours after burn injury, reaching a minimum at 72–120 hours, correlated to the size of the burn injury and potentially serving as an early indicator for concurrent infections. Further characterization of neutrophil chemotaxis using this new assay may have important diagnostic implications not only for burn patients but also for patients afflicted by other diseases that compromise neutrophil functions.

## Introduction 

In the United States, 40,000 patients are hospitalized with severe burn injury annually[1]. Although goal-directed resuscitation [2]–[4], early burn wound excision and grafting [5]–[8], and recognition of burn hypercatabolism [9]–[11] have dramatically improved outcomes for these patients, the mortality rate from burn injury has changed little over the past twenty years, hovering at 5–10% [1], [12]. Among factors responsible for patient outcomes, sepsis delivers the most profound impact, and contributes to 75% of deaths [13], [14]. Additionally, sepsis poses special diagnostic challenges in the burn population, whose chronic inflammatory state renders the usual clinical signs of infection – leukocytosis, fever, and tachycardia –nonspecific and unreliable [15]. The critical influence of infection on patient outcomes, as well as the diagnostic difficulties that arise in the burn population, call for new modalities in the identification and characterization of sepsis in burn patients.

Multiple studies have documented impairments in neutrophil function after burn injury, with potential implications for the development of sepsis [16]–[23]. Flow cytometry analysis of neutrophils reveals impairments in phagocytosis, bactericidal activity, phago-lysosomal activity, and the oxidative burst within two weeks post-injury [24]. Neutrophils demonstrate impaired adhesion and complement receptor expression after burn injury [25]–[31], and these changes, in turn, correlate with an increased incidence of abscess formation *in vitro* [32]. In 1975, Warden *et al.* evaluated the chemotaxis function of neutrophils from 46 burn patients with a Boyden chamber assay, and compared the results with those of 44 healthy volunteers [17]. In all cases, patients had defective chemotaxis indexes and, within 72 hours after burn injury, the degree of impairment correlated with burn size and was predictive of mortality from septic complications [17], [33]. One limitation of this and other studies using the Boyden assay is the lack of direct observation of the moving cells. The outcome of the Boyden assay is a chemotactic index that depends on the size of initial cell population, the fraction of cells that moves, and their speed, directionality, and persistence. Consequently, these studies could not identify the source of the changes in neutrophil chemotaxis after burn injuries, or if one or more the chemotaxis parameters have changed at the same time. More detailed analysis using Zigmond assay that allows the examination of chemotaxis by direct observation of individual neutrophils, suggested that directionality of migration but not migration speed correlate with the overall magnitude of burn injuries [18]. Overall, despite evidence suggesting a link between neutrophil chemotaxis and outcomes after burn injury, existing assays for neutrophil migration are difficult to implement in the clinical setting, and as such, the prognostic potential of chemotaxis measurements remains largely unexplored.

Recent advances in microfluidic technologies provide new opportunities for cell based assays for clinical research applications, ranging from AIDS diagnostic [34], to trauma [35] and cancer monitoring [36]. Specifically for chemotaxis applications, after the first reported neutrophil chemotaxis measurements with a microfluidic device [37], more devices for analyzing neutrophil migration in response to opposing chemoattractant gradients [38], temporal changes of the gradients [39], radialy evolving gradients [40], and overlapping spatial stimuli [41] have been reported. A broad range of applications, from the study of unexpected neutrophil ability to migrate in certain conditions against chemoattractant gradients a.k.a. fugetaxis [42], to practical devices for on-chip isolation of neutrophils from a drop of blood [43], and testing of new compounds modulators of inflammation response [44] were enabled by the use of microfluidic devices. Unfortunately, while these microfluidic assays have permitted sophisticated experimentation in the laboratory, they are difficult to implement in the clinical setting. New designs are needed to overcome practical obstacles, like the requirement for expensive syringe pumps or specialized training of the operator.

To address the need for a robust, yet practical assay to investigate the details of neutrophil chemotaxis in burn patients, we designed a no-flow microfluidic device to measure the directional migration speed in chemoattractant gradient, with high throughput, and at single cell resolution. We validated the device by measuring the migration speed of neutrophils in blood samples from healthy volunteers and established one reference value for healthy persons regardless of age and sex. We also employed this device to document the impairment of neutrophil migration speed after burn injury with a degree of precision previously unattainable. We found that neutrophil motility is depressed as early as 24 hours after the burn injury and is inhibited the most at three to five days after injury. We found that the degree of neutrophil directional migration speed inhibition in burn patients correlates with the magnitude of burn trauma. Surprisingly, the migration speed did not correlate with clinical parameters frequently used to monitor these patients, e.g. absolute neutrophil numbers, age of circulating neutrophils, or the body temperature. These results suggest more complex relationships between the impaired neutrophil chemotaxis and the clinical evolution of the burn patients could be uncovered in further studies.

## Methods

### Fabrication of the microfluidic devices

Microfluidic devices used to measure neutrophil directional migration speed in response to chemoattractant gradients were manufactured using standard techniques. Two layer of photoresist (SU8, Microchem, Newton, MA), the first one 3 µm thin and the second one 50 µm thick, were patterned on one silicon wafer by sequentially employing two photolithography masks and processing cycles according to the instructions from the manufacturer. The wafer with patterned photoresist was used as a mold to produce PDMS (Polydimethylsiloxane, Fisher Scientific, Fair Lawn, NJ) parts, which were then bonded irreversibly to standard glass slides (1×3 inches, Fisher). Up to six devices were bonded side by side on one glass slide and transported to the clinic in sealed Petri-dishes.

### Sample preparation

Blood samples of 1 mL were obtained from healthy volunteers, aged 18 years and older, who were on no immuno-suppressants. A maximum of three samples were drawn from each volunteer, with a time lapse of at least one week between draws. Additionally, blood samples of 1 mL were drawn from burn patients admitted to the Massachusetts General Hospital (MGH) and Shriners' burn units. Patients were enrolled if they sustained burns covering at least 20% of their total body surface area. The first sample was obtained within 72 hours after burn injury, and two more samples were drawn at 48 hour intervals afterward. No sample was drawn within 24 hours of an operative procedure. All patient samples were obtained with written informed consent, and through procedures approved by the MGH and Shriners Institutional Review Boards.

### Neutrophil isolation

Using sterile technique, neutrophils were isolated from whole blood by density gradient separation using Polymorphprep (13.8% sodium diatrizoate and 8.0% polysaccharide, Axis-Shield, Rodelokka, Oslo, Norway), with centrifugation at 500 *g* for 40 minutes. To return the cells to an isotonic environment, they were harvested and re-suspended in 10 mL of 0.5× PBS, then isolated by centrifugation at 400 *g* for 10 minutes. The final aliquot of neutrophils were re-suspended in 50–100 µL of 1× PBS before loading into the chemotaxis device. Samples were processed within one hour after each blood draw, and were maintained at 37°C.

### Neutrophil chemotaxis measurements

**Figure 1:**
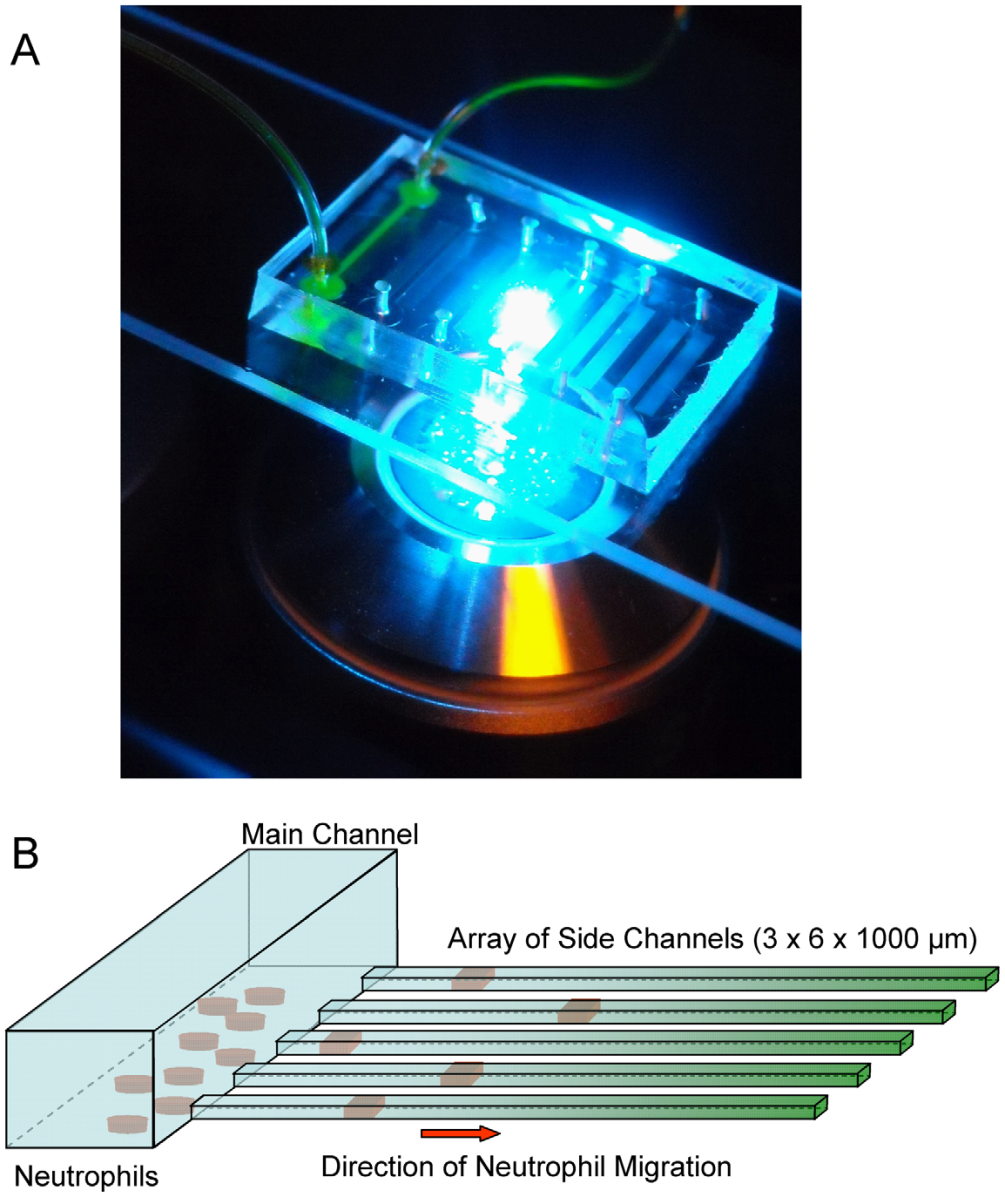
Overview of the neutrophil chemotaxis device. **a**. Microfluidic devices mounted on glass slides are observed on a microscope stage. In this picture, six chemotaxis devices are aligned side by side on the same glass slide. The left most device has inlet and outlet tubing attached and is filled with green fluorescent dye. **b**. Schematic of the microfluidic device. The device consists of a larger main channel and an orthogonal array of smaller channels. In the first step, the whole device, including the side channels, is primed with the chemoattractant solution. In the second step, neutrophils suspended in buffer are introduced in the main channel, washing out the chemoattractant from the main channel. The chemoattractant gradient is established by diffusion, in the longitudinal direction of the side channels, between the end of the side channels filled with chemoattractant and the main channel filled with buffer. Neutrophils inside the main channel follow a chemoattractant gradient and enter an array of side channels, where their chemotaxis migration speed is measured.

15 minutes before neutrophils were loaded, the microfluidic device was primed with the chemokine fMLP [100 nM] and the extracellular matrix protein fibronectin [100 µM]. For this, a 1 mL syringe filled with the solution of fMLP and fibronectin was connected to one port of the device, and the outlet port was blocked. By applying pressure to the syringe, the solution was instilled into the device, and the displaced air diffused out through the PDMS. Neutrophils were then infused into the device and allowed to settle in the main channel by clamping the ports of the device. Neutrophil migration in the direction of chemoattractant started immediately, and was recorded using and time-lapse imaging and 20× magnification on a Zeiss Axiovert microscope (**Fig. 1a**). At least 50 neutrophils were manually tracked in each sample, and velocities were calculated using Image J (NIH). Separate experiments to characterize the formation of gradients inside the device in the absence of neutrophils were performed by replacing the fMLP with fluorescein (Sigma) of comparable molecular weight, and analyzing the distribution and changes in fluorescence intensity from time-lapse imaging.

### Statistical analysis

To test the normality of the distribution of migration speed values for neutrophils in the same sample we employed the Shapiro-Wilk test, indicating if the data is likely to be derived from a normally distributed population (p>0.05). Correlations between neutrophil migration speed and clinical parameters were calculated using multivariate analysis.

## Results

**Figure 2:**
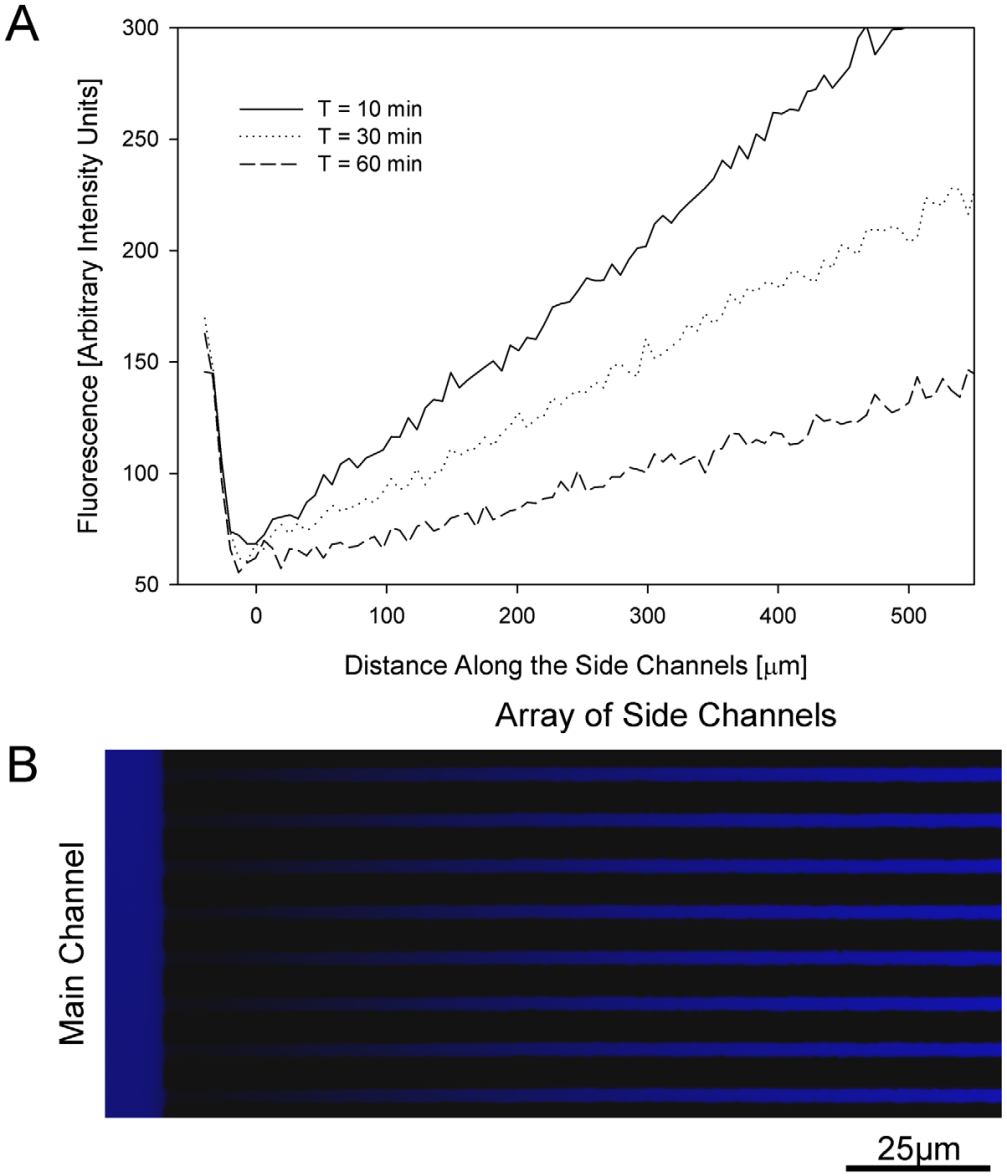
Visualization of the chemical gradients along the side channels. **a**. Linear gradients, formed along the side channels, were imaged using fluorescent dyes of molecular weight comparable with that of the chemoattractant. Starting immediately after replacing the solution in the main channel with buffer, quantitative fluorescence was measured along the array of side channels at 10, 30 and 60 minutes. These measurements demonstrate the relative stability of the linear chemoattractant gradient over time. **b**. At 30 minutes after introducing the buffer, a linear gradient of fluorescent dye can be visualized along the side channels. The height of the main channel (to the left) is larger that that of the side channels and accounts for the apparent higher fluorescence of the main channel compared to the side channels.

To measure neutrophil migration speed in response to soluble chemoattractants, we designed a microfluidic device that establishes chemokine gradients between a central, buffer-filled channel that acts as a sink, and an array of smaller side channels that act as chemoattractant reservoirs. The side channels communicate with the central channel at one end, and are closed at the distal end (**Fig. 1**). Priming the device with chemoattractant fills these side channels. During infusion of neutrophils into the device, the buffer solution in which neutrophils are suspended replaces chemokine from the central channel. At the same time, most of the chemoattractant in the array of side channels remains in these channels secondary to their high length to cross section area ratio. When the flow of buffer and neutrophils ceases, progressive diffusion of the chemoattractant establishes a gradient in the longitudinal direction of the side channels (**Fig. 2**). Neutrophils from the central channel will follow this gradient and enter the side channels. Moving neutrophils are mechanically confined in the 6 µm width, 3 µm height channels, and consequently, neutrophils obstruct the channels and trap chemoattractant distally, creating steep differences in stimulation in front of and behind the moving neutrophils.

### Device validation

**Figure 3:**
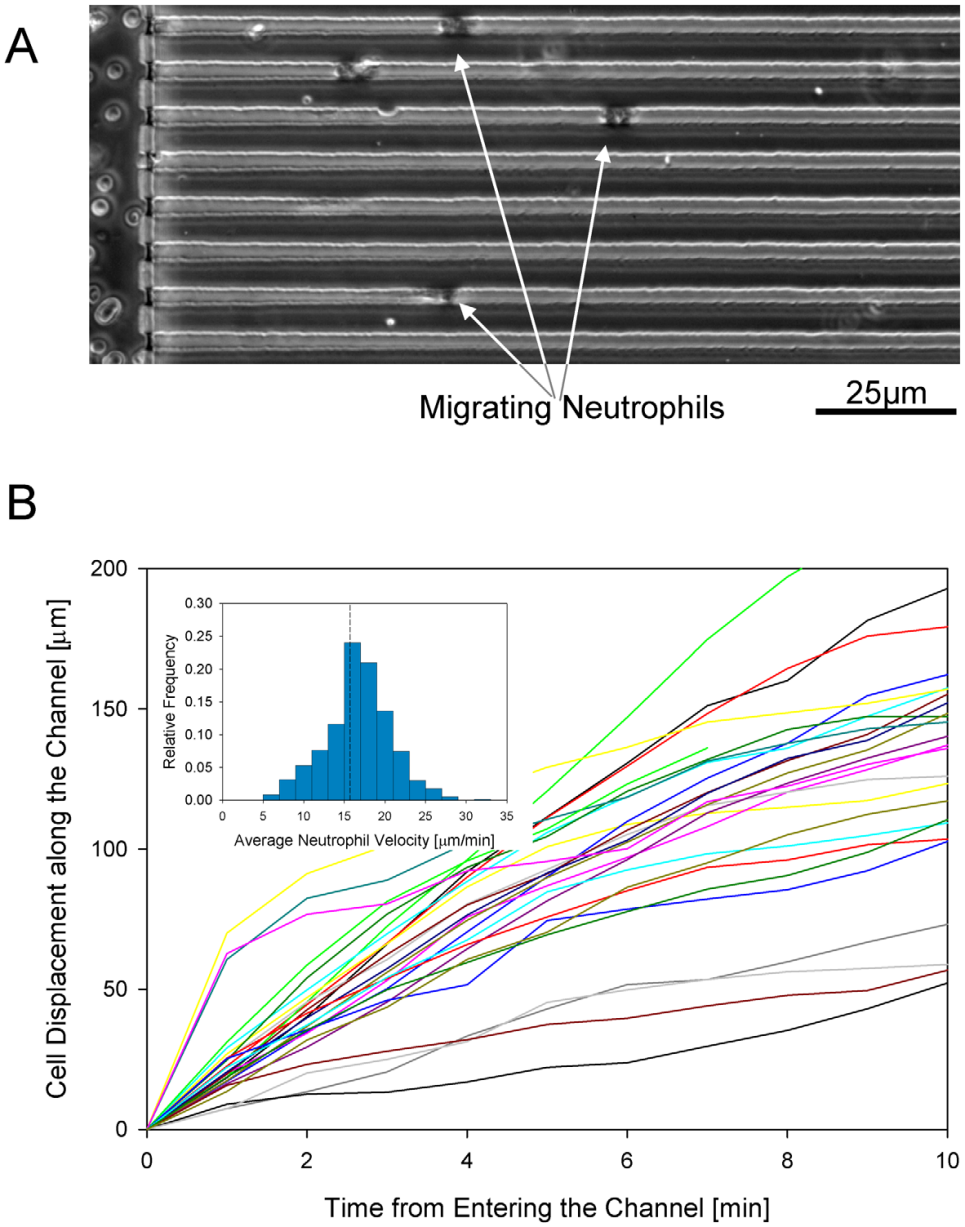
Neutrophil migration inside the array of channels. **a**. Neutrophils enter the array of channels and move along the channels towards higher concentrations of chemoattractant – see also **Movie S1**. **b**. Displacement of neutrophils vs. time inside the array of channels. For the first 10 minutes after entering the side channels neutrophil display remarkably uniform migration speed. Insert shows the distribution of average speed of migration for 800 neutrophils from one healthy donor.

Most of the neutrophils maintain constant speed toward fMLP within the channels for at least the first 10 minutes (**Fig. 3** and **Movie S1**). Interestingly, a few neutrophils moved faster for the first 100 µm inside the channels, decelerated later, and maintained constant speed thereafter (**Fig. 3b**). Moving neutrophils displayed persistent behavior along the channels, with no changes in the direction of migration. In one experiment, we calculated the average speed of all cells and verified the normality of distribution around the mean of the velocities of the almost 800 cells moving through the side channels (**Fig. 3b**, insert). We also compared this with the average migration speed of 50, 100 and 200 cells randomly chosen at different locations throughout the device. We observed no statistically significant difference between the four populations and in subsequent experiments we analyzed only 50 moving neutrophils, at random locations throughout the device. In additional control experiments, in the absence of fMLP chemokine, neutrophils did not migrate through the side channels of the device. Furthermore, when a sample of neutrophils was diluted ten-fold, the measured average migration speed was unchanged (17±6 µm/min and 18±4 µm/min, *p* = 0.3), suggesting that migration speed was independent of the concentration of neutrophils in the device.

### Neutrophil migration in blood samples from healthy donors

**Figure 4:**
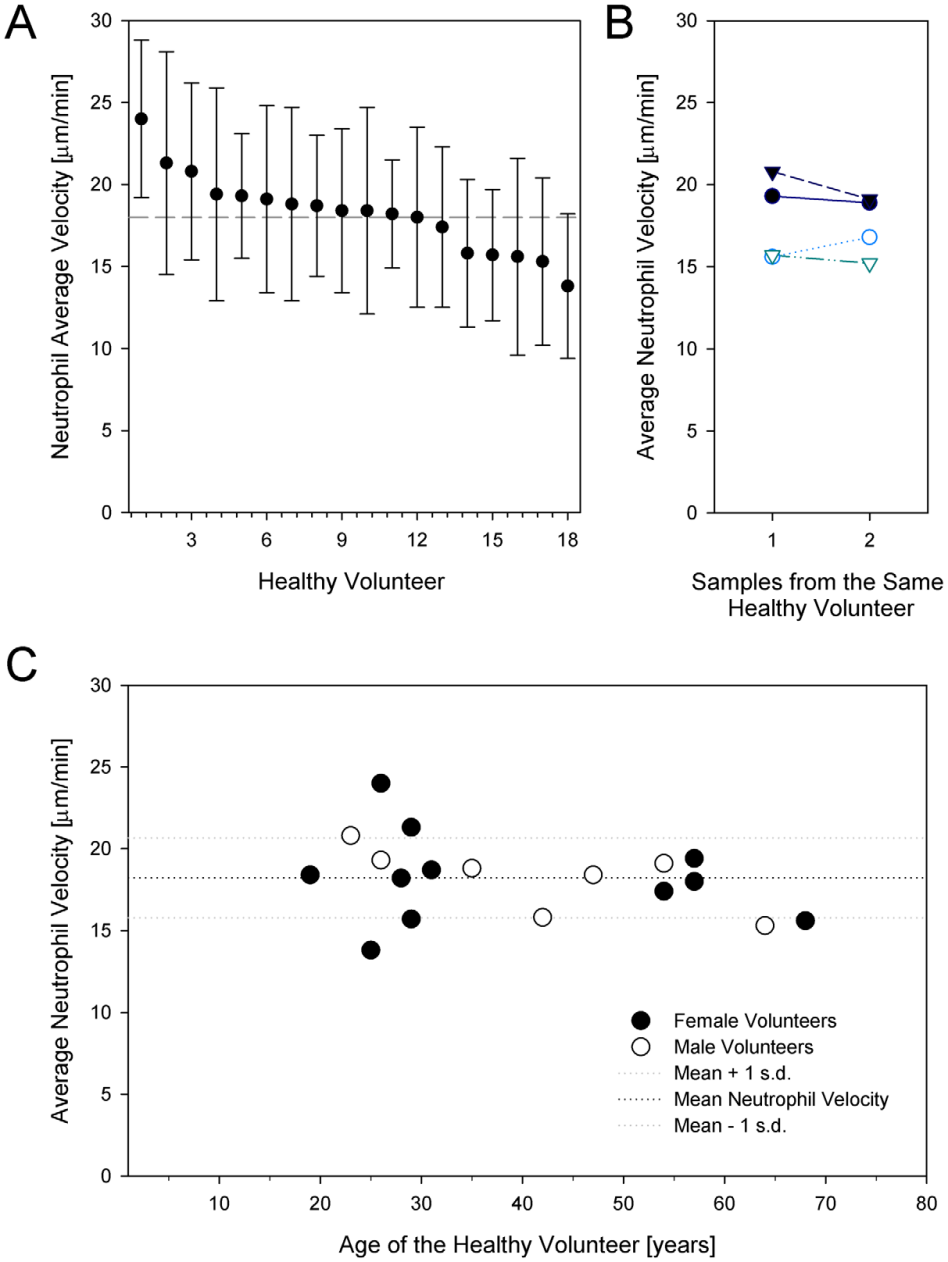
Neutrophil motility in healthy donors. **a**. Average neutrophil motility in 18 healthy donors. Samples were rearranged in decreasing order of the average motilities. Bars represent standard error of the mean. The motility of at least 50 neutrophils was calculated for each sample. **b**. Validation of the repeatability of neutrophil motility. Two samples from the same healthy donors were collected at two weeks time interval and neutrophil migration speed measured in the microfluidic devices. **c**. Distribution of average values for neutrophil motility with the age of healthy donors. No significant changes in neutrophil motility were observed with increasing age of the healthy volunteers. No significant differences were observed between female and male donors (filled and empty dots, respectively).

A total of 23 blood samples were collected from 18 healthy volunteers aged 19–68 years (mean 40 years) (**Table 1**); 61% of volunteers were female, and 39% male. Volunteers had no past medical history with the exception of hypertension, hypothyroidism, and depression. The most common medications taken were oral contraceptives and antidepressants. No volunteer took immunosuppressant drugs. Average neutrophil migration speed for all volunteer samples was 18±5 µm/min (range 14 to 24 µm/min) (**Fig. 4a**). This average is within the limits of previously reported values for normal neutrophil speed measured by standard techniques [17]. To verify the reproducibility of our neutrophil migration speed measurements, we repeated experiments with neutrophils isolated from 4 healthy volunteers, with samples drawn between one week and one month apart. The experiment yielded equivalent neutrophil velocities, thus suggesting high reproducibility (**Fig. 4b** **,** *p*≥0.05 in all cases). There were no correlations between the neutrophil migration speed and the volunteer age or gender (**Fig. 4c**).

**Table 1:** Clinical overview of burn patients' data.

	Volunteers	Patients
**No. Participants**	18	8
**Average Age in Years (Range)**	40(19–68)	31(1–56)
**Female** **(%)**	11(61)	2(25)
**Male** **(%)**	7(39)	6(75)
**Caucasian** **(%)**	17(94)	5(63)
**Hispanic** **(%)**	0	3(37)
**Asian** **(%)**	1(6)	0
**No. without Comorbidities (%)**	10(56)	5(63)
**Comorbidities** **(in descending order of frequency)**	DepressionHyperlipidemiaHypertensionHypothyroidism	DepressionHyperlipidemiaHypertensionCirrhosis
**Medications** **(in descending order of frequency)**	Oral contraceptivesSSRIsACE InhibitorsDiureticsThyroid replacement therapyStatinsProton pump inhibitors	ACE InhibitorsBenzodiazepines
**No. with Inhalation Injury** **(%)**	N/A	3(13)
**No. Requiring Mechanical Ventilation (%)**	N/A	13(54)
**No. with Positive Blood Cultures <72 hrs Post-Burn (%)** **-Microbes-**	N/A	1(13)*-Acinetobacter-*
**No. with Positive Blood Cultures 1–4 Weeks Post-Burn (%)** **-Microbes-**	N/A	4(50)-*Enterococcus, MRSA, MSSA, Enterobacter, Candida-*
**No. with Positive Sputum Cultures <72 hrs Post-Burn (%)** **-Microbes-**	N/A	2(25)*-MSSA, H. influenzae-*
**No. with Positive Sputum Cultures 1–4 Weeks Post-Burn (%)** **-Microbes-**	N/A	2(25)-*E. coli, MRSA-*

### Neutrophil migration in blood samples from burn patients

**Figure 5:**
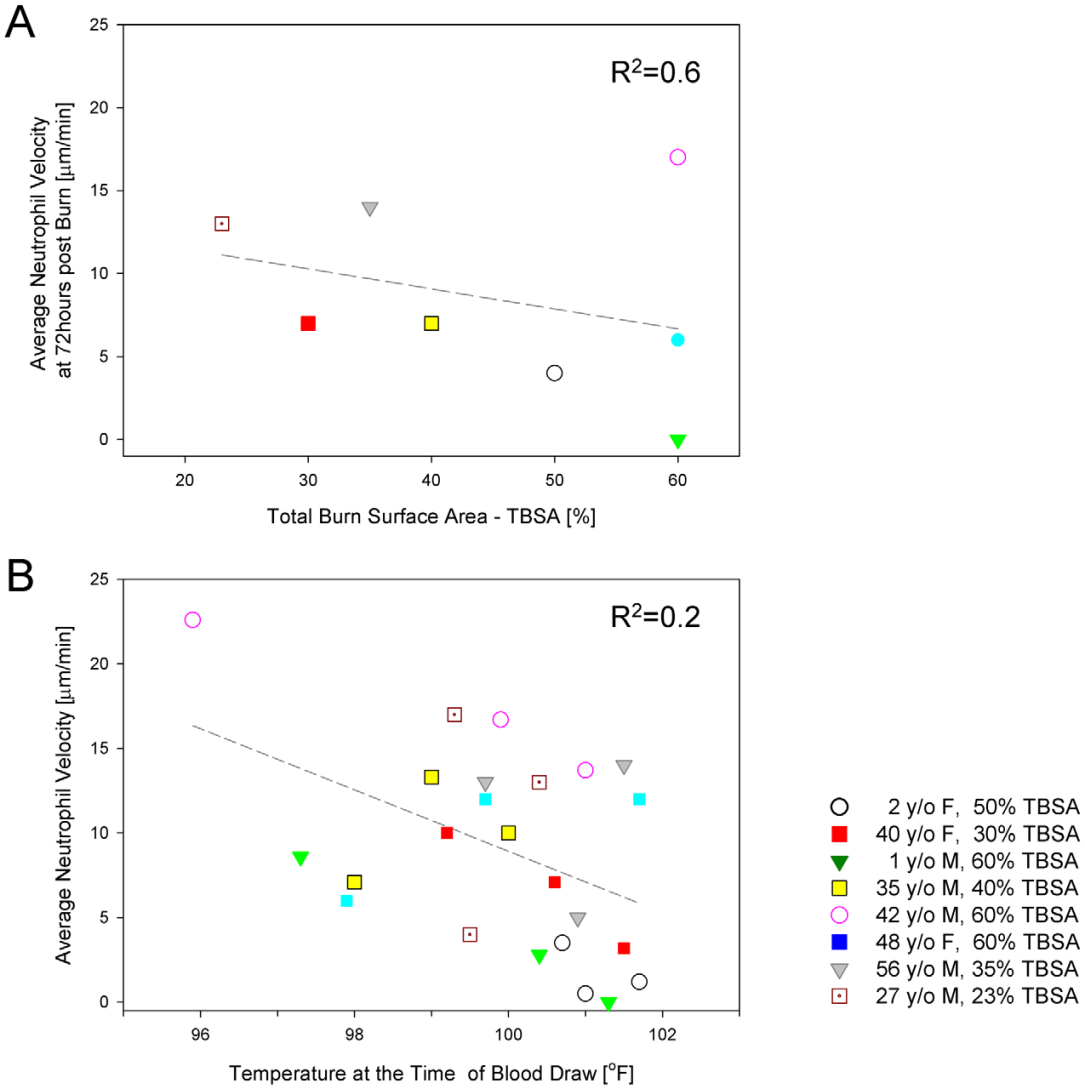
Correlations between neutrophil motility and clinical parameters in burn patients. **a**. We observed significant correlations between neutrophil motility at 72 hours after burn injury and total burn surface area (R^2^ = 0.6). **b**. No significant correlation was found between neutrophil motility and the temperature in burn patients (R^2^ = 0.2). Samples from the same patient are coded using the same symbol.

**Figure 6:**
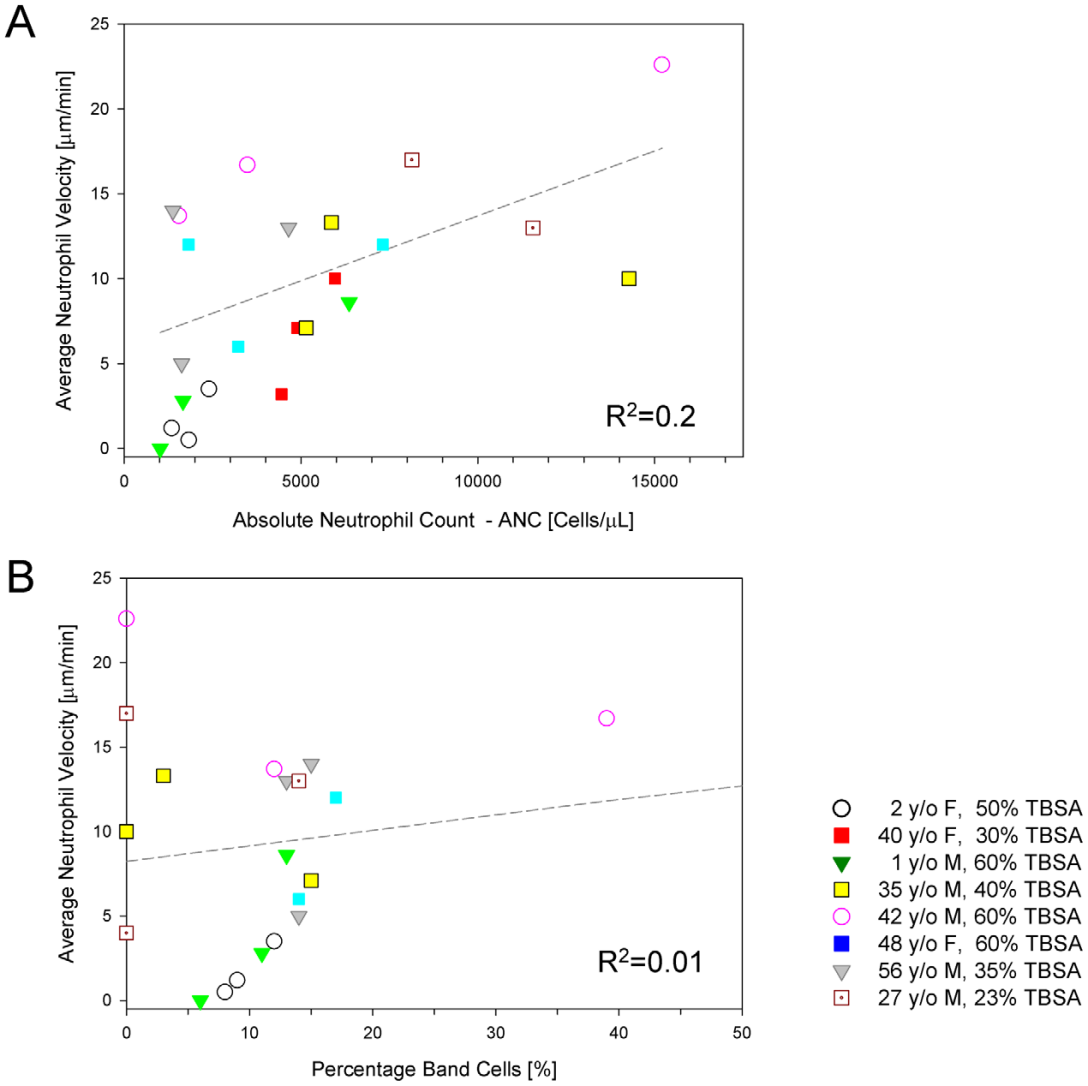
Correlations between neutrophil motility and neutrophil counts in burn patients. **a**. No significant correlation was found between neutrophil motility and the absolute neutrophil count (R^2^ = 0.2). **b**. Also, there was no significant correlation between average neutrophil motility and percentage of band cells in the neutrophil population (R^2^ = 0.01).

**Figure 7:**
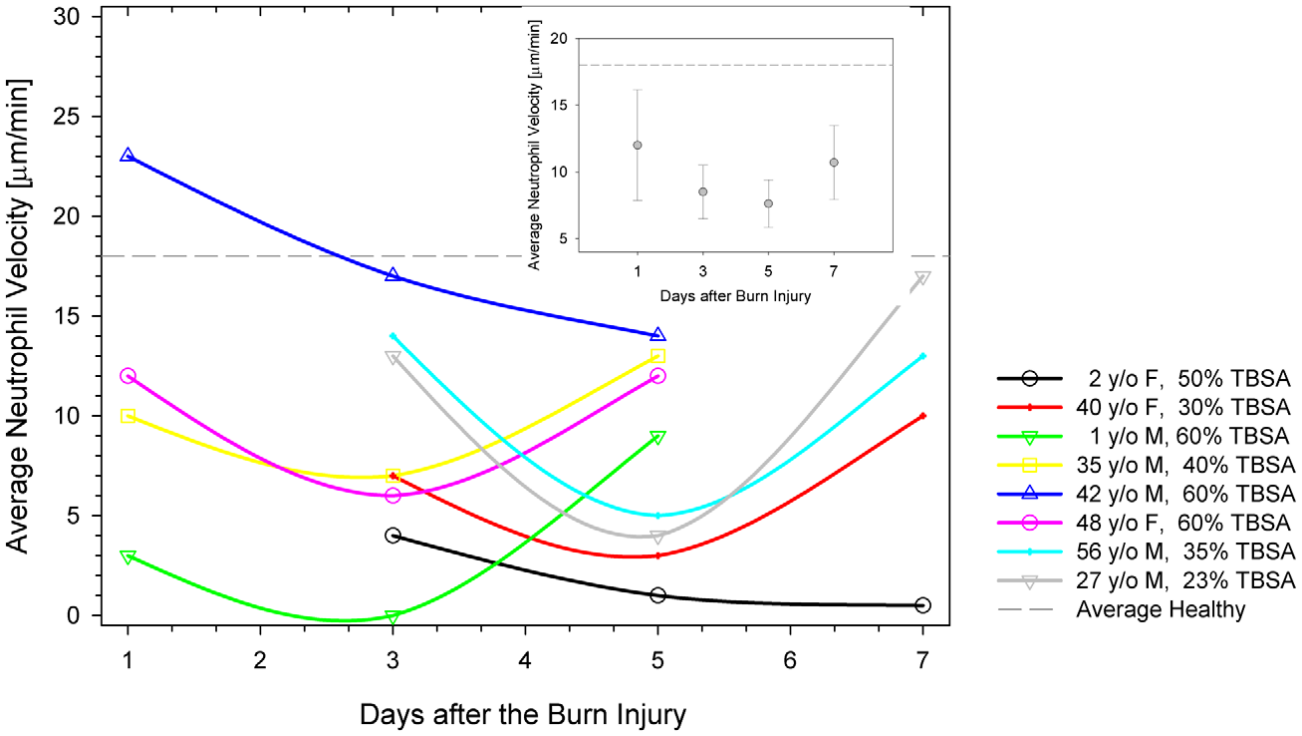
Changes in neutrophil motility in burn patients. Average neutrophil motility in individual patients was measured at 48 hours intervals after admission to the hospital. Lower values for neutrophil migration speed compared to healthy volunteers (dashed gray line) were recorded as early as 24 hours after burn injury for the majority of the patients. Insert shows the changes in average neutrophil migration speed for all patients, with respect to the time of injury.

Neutrophil motility was measured in a total of 24 samples from 2 pediatric and 6 adult patients (mean age 23 years, range 1–48 years) after sustaining 20–60% TBSA burns (**Table 1**); 63% of patients were male, and 38% were female. The most common co-morbidities were depression and hypertension. No patient took immunosuppressant drugs. Across the entire data set, the average neutrophil migration speed in burn patients was 9±6 µm/min, significantly lower than in controls (*p*<0.01). In patients who were not bacteremic at 72–120 hours post-injury, neutrophil migration speed was negatively correlated with severity of burn injury (**Fig. 5a**, R^2^ = 0.6). Across the entire data set for burned patients, there was poor correlation between neutrophil migration speed and temperature (R^2^ = 0.2) (**Fig. 5b**), absolute neutrophil count (R^2^ = 0.23), or the percentage of band cells in the neutrophil population (R^2^ = 0.01) (**Fig. 6**). Neutrophil motility was most depressed between 72 and 120 hours after injury (**Fig. 7**), with an average migration speed of 8±2 µm/min. After this time frame, neutrophils demonstrated a tendency toward motility recovery, with an average migration speed of 11±3 µm/min one week after the burn injury. One exception to this pattern was a 42 year-old male who sustained 60% TBSA burns, who had positive sputum cultures for *Haemophilus influenzae* and blood cultures positive for *Acinetobacter* at the time his neutrophils were analyzed. No other patient had documented bacteremia at the time of enrollment. A second exception was a 2 year-old female patient who sustained 52% TBSA flame burns, whose neutrophil motility continued to decline at the third blood draw. This patient had persistently high fevers for up to two weeks after her initial burn injury, prompting institution of empiric antibiotics. Despite broad antibiotic coverage, she developed bacteremia with *Enterococcus* three weeks after her initial insult.

## Discussion

We have developed and validated a microfluidic device for the quantitative analysis of neutrophil directional migration speed in the clinical setting. The device utilizes small volumes of blood, a practically important feature to avoid iatrogenic anemia by repeated blood draws from critically ill or pediatric patients. The assay yields data in less than three hours, and requires only the sequential injection of two solutions through the same port of the device, without syringe pumps or complicated priming. The assay provides temporal information at the single-cell level, and while most of the neutrophils migrate at steady speed along distinct channels of our device, their migration speed can be measured to a degree of precision difficult to attain by standard methods. A comparison between this assay and other techniques based on traditional or microfluidic technologies, is presented in **Table 2**. For example, the Boyden chamber [45], which for decades served as the standard laboratory technique for evaluating chemotaxis, can only provide an overall measure of the chemotaxis function and cannot quantify the contribution of changes in migration speed, persistence or directionality to the eventual changes of the chemotaxis function in various context. While being easy to use, it also requires large numbers of cells, separated from several milliliters of blood, disallows direct visualization of individual cells, and cannot differentiate between chemotaxis and random cell motility. More sophisticated assays, such as the Zigmond and Dunn chambers [46], [47], permit examination of individual cells motility and are frequently used in biology laboratories. However, the setup and use of these chambers are labor intensive, require expertise and do not translate easily to the bedside. Recently, microfluidic devices have higher precision of the chemoattractant gradients forming inside [37]–[39]. These assays require only small number of cells, translated into the need for smaller blood samples, but their use relies on external pumps and trained operators. Like other assay where neutrophils move on flat surfaces [37], [39], [42], [46]–[51], they also require parametric analysis of the “biased random walk” migration pattern characteristic of neutrophils [52]. While the gradients in our device are not as stable as in other microfluidic flow devices [37], neutrophils moving inside the small side channels of are exposed to steeper differences in chemoattractant concentrations, which could partially explain the observed robust chemotaxis in such devices [53]. Finally, the mechanical confinement of neutrophils in small channels in this and other devices [53], [54], appears to facilitate the persistent movement and has practical consequences the ease of quantification of motility.

**Table 2:** Comparison of technologies for measuring neutrophil chemotaxis.

	Single Cell Resolution	Small Sample Size	Ease of Use	Pumps Required	References
**Traditional Assays**					
Transwell	-	-	yes	-	[45]
Micropipette	yes	yes	-	yes	[49]
Dunn, Zigmond Chambers	yes	yes	-	-	[46], [47]
Under-Agarose	-	-	yes		[60]
**Microfluidic Assays**					
Gradient under Flow	yes	yes	-	yes	[37]–[39], [42]–[44], [50], [51], [61]
Diffusion Chambers	yes	yes	-	-	[40], [48], [62]–[64]
Convection-Free Channels	yes	yes	-	yes	[41], [53], [54], [65], [66]
**This assay**	yes	yes	yes	-	-

Using the new assay, we established a normal range of neutrophil directional migration speed values for healthy volunteers. Our volunteer studies using the device have yielded an average neutrophil migration speed toward chemoattractant of 18 µm/min, establishing a benchmark value for neutrophil motility towards fMLP in healthy persons. The average neutrophil migration speed was in accordance with data from standard chemotaxis assays. We also found no dependence of the average neutrophil speed on the sex or age of the donor. Moreover, we documented the reproducibility of the measurements by repeating the experiments in four of the volunteers at one- to two-week time intervals. Together, these results establish the assay as a valuable tool for measuring neutrophil motility, and provide a reference set of values characteristic of neutrophils from healthy subjects.

The assay is sensitive enough to detect significant impairment of neutrophil migration speed as early as 24 hours after patient admission to the hospital. This finding is new, and earlier studies using traditional assays did not report any changes in neutrophil chemotaxis until 72 hours e.g. Solomkin et al using an under-agar assay [22], Chalain et al using an variation of Boyden assay [55], Kim et al using a Zigmond assay [18]. The ability to measure precisely the neutrophil migration speed independently of all the other parameters of chemotaxis, also allowed us to establish a strong correlation between the depression of migration speed at 72 hours after burn correlated and the total body surface area (TBSA) of burn injury. In contrast, earlier studies have only been able to identify correlations between the chemotaxis index as measured by the Boyden chamber and TBSA [16], and at least one other study found no correlation between TBSA and motility [18]. One source for the differences could have been the fact that most current assays measure a global chemotaxis index that depends on multiple aspects of neutrophil migration, including the fraction of the neutrophil population that are able to move, the alterations of speed, directionality or persistence of migration towards chemoattractant gradients. Together, these new findings enabled by the microfluidic assay could contribute to better understanding of the context of neutrophil pathology associated with the burn injury and its contribution to the septic and other complications in burn patients.

One more interesting observation in our study was the correlation between the timing of most pronounced depression of neutrophil migration speed that was measured at 48 hours after the first therapeutic measure. One could speculate on the temporal relationship between the aggressive fluid resuscitation, common among all patients, which was started in the days and hours preceding the first blood draw, with a total body balance in excess of liters. By the second blood draw in all cases, resuscitation efforts had concluded, and patients received crystalloid infusions at one- to two-times their maintenance rate (as calculated by ideal body weight). The immuno-modulatory effects of massive crystalloid resuscitation have been described and large-volume infusions of isotonic fluids were found upregulate the neutrophil oxidative burst, increase expression of adhesion molecules, and induce cellular injury [56]. Inappropriate activation of neutrophils after crystalloid administration, evaluated *in vitro*, has been hypothesized as a possible mechanism for ARDS and end-organ injury in trauma patients [56]. Studies have also established increased rates of post-operative infection and colon cancer recurrence among patients who receive transfusions perioperatively [57] suggestive for transfusion-related immunosuppression. Despite these effects, few data exist on individual neutrophil responsiveness to chemoattractant after crystalloid resuscitation. The depression in neutrophil migration speed observed among this cohort of patients may provide a window into the possible link between crystalloid resuscitation and neutrophil migration.

A key goal for further studies will be to establish if neutrophil motility is a more reliable diagnostic or prognostic indicator of infection among burn patients than the nonspecific parameters of white blood cell count and fever. Despite the profound role that sepsis plays in the morbidity and mortality of burn patients, standard clinical and laboratory markers of infection are unreliable in the setting of severe burn injury [58]. For most clinical conditions, fever, leukocytosis, tachycardia, increased respiratory rate, and hypotension signal the onset of sepsis. In the burn population, however, the massive inflammatory cascade that follows thermal injury, coupled with insensible volume losses, trigger these findings even in the absence of infection. The dilemma is so profound that in 2007, the American Burn Association published a consensus statement that condemned the peripheral white blood cell count as an appropriate diagnostic criterion for sepsis in burn patients [15]. Our preliminary results suggest that preservation of neutrophil chemotaxis in burn patients may correspond with bacteremia, and may signal the need for antibiotic therapy in the absence of culture data. All but one patient demonstrated depressed neutrophil motility compared with controls, with maximal depression at 3–5 days post-injury. Of these patients, most (60%) developed delayed infection, i.e., pneumonia or bacteremia several weeks after analysis. Those patients who did not have delayed infection documented by culture data, were still treated empirically with antibiotics for suspected infection. The one patient whose motility did not follow this pattern, and who in fact maintained supra-normal to normal neutrophil migration speed, was a 42 year-old male with 60% TBSA burns who had positive blood and sputum cultures on admission to the burn unit. He was the only patient who had documented infection by culture data at the time of neutrophil analysis. Based on this preliminary data, it is possible that maintenance of normal neutrophil motility in the acute phase after burn injury corresponds with active bacterial infection. As cultures require at least 48 hours for results, neutrophil motility may provide an earlier confirmatory marker of sepsis than current methods. This potential would be particularly useful for targeting patients who require antimicrobial therapy during in an era when resistance of microbes to pharmacologic agents continues to escalate.

In conclusion, we designed a microfluidic device that allows for robust measurements of neutrophil motility at single-cell resolution. We applied this device to neutrophils isolated from healthy volunteers and established a range of normal values for neutrophil migration. We further validated the new technique for measuring neutrophil chemotaxis speed through repeated experiments. When we applied the technique to neutrophils from burn patients, we documented a significant reduction in average neutrophil migration speed and the dynamic evolution of neutrophil migration impairment. The ability to screen large numbers of neutrophils at single-cell resolution could allow for the study of cell phenotype under various clinical circumstances. Simple comparisons between patients and the healthy average could provide insight into management of immune diseases that alter neutrophil function via point-of-care microfluidic devices. Modulation of the immunosuppressant regimen in transplant recipients, risk of infection among chemotherapy patients, influence of hemorrhage and massive transfusion after trauma, pathophysiology of Chediak Higashi syndrome and chronic granulomatous disease [59], or cellular changes in sepsis and ARDS, all represent potential avenues of clinical research to which chemotaxis data may contribute. Furthermore, quantitative analysis of neutrophil chemotaxis could optimize recommendations for immuno-modulatory and antibiotic therapies, and ultimately improve outcomes in patients suffering from severe burn injury and other immune-suppressed states.

## Supporting Information

Movie S1 Human neutrophils migrating inside the array of channels. Neutrophils, isolated from a healthy volunteer, enter the array of channels and move towards higher concentrations of chemoattractant fMLP [100 nM]. Individual images recorded every minute for ten minutes were combined in a time-lapse movie showing the migration of three representative neutrophils through the channels. The width of the channels is 6 µm.
(2.04 MB AVI)
